# Transcriptomic profile induced by calcitriol in CaSki human cervical cancer cell line

**DOI:** 10.1371/journal.pone.0319812

**Published:** 2025-04-01

**Authors:** Euclides Avila, Luis David Hernández-Monterde, Alberto Cedro-Tanda, Tomas Misael Lizardi-Aguilera, David Barrera, Francisco Vladimir Villegas-Rodriguez, Janice García-Quiroz, Lorenza Díaz, Fernando Larrea

**Affiliations:** 1 Departamento de Biología de la Reproducción Dr. Carlos Gual Castro, Instituto Nacional de Ciencias Médicas y Nutrición Salvador Zubirán, Mexico City, Mexico; 2 Instituto Nacional de Medicina Genómica, Mexico City, Mexico; Ege University Faculty of Medicine: Ege Universitesi Tip Fakultesi, TÜRKIYE

## Abstract

The vitamin D endocrine system, primarily mediated by its main metabolite calcitriol and the vitamin D receptor (VDR), plays a critical role in numerous human physiological processes, ranging from calcium metabolism to the prevention of various tumors, including cervical cancer. In this study, we comprehensively investigated the genomic regulatory effects of calcitriol in a cervical cancer model. We examined the transcriptional changes induced by calcitriol in CaSki cells, a cervical cell line harboring multiple copies of HPV16, the primary causal agent of cervical cancer. Our microarray findings, revealed that calcitriol regulated over 1000 protein-coding genes, exhibiting a predominantly repressive effect on the CaSki cell transcriptome by suppressing twice as many genes as it induced. Calcitriol decreased EPHA2 and RARA expression while inducing KLK6 and CYP4F3 expression in CaSki cells, as validated by qPCR and Western blot. Functional analysis demonstrated that calcitriol effectively inhibited key processes involved in cancer progression, including cell proliferation and migration. This was further supported by the significant downregulation of MMP7 and MMP13 mRNA levels. Our microarray results also showed that, in addition to its effects on protein-coding genes, calcitriol significantly regulates non-coding RNAs, altering the expression of approximately 400 non-coding RNAs, including 111 microRNA precursors and 29 mature microRNAs, of which 17 were upregulated and 12 downregulated. Notably, among these calcitriol-regulated microRNAs are some involved in cervical cancer biology, such as miR-6129, miR-382, miR-655, miR-211, miR-590, miR-130a, miR-301a, and miR-1252. Collectively, these findings suggest that calcitriol exhibits a significant antitumor effect in this advanced cervical cancer model by blocking critical processes for tumor progression, underscoring the importance of maintaining adequate vitamin D nutritional status.

## Introduction

Cervical cancer, predominantly affecting middle-aged women in underdeveloped regions, ranks as the fourth most prevalent cancer in women globally, following breast, lung, and colorectal cancers [1]. Persistent infection with high-risk strains of human papillomavirus (HPV), particularly types 16 and 18, stands as the primary etiological determinant of cervical carcinogenesis [2]. However, multiple additional factors contribute to the pathogenesis of cervical cancer [3]. Emerging evidence suggests a potential link between vitamin D deficiency and increased cervical cancer risk, indicating a protective role of vitamin D against this malignancy [4].

Calcitriol, the most potent metabolite of vitamin D_3_, is synthesized through two sequential hydroxylation steps. The first step occurs on vitamin D_3_, and the second on 25-hydroxyvitamin D_3_. These processes occur in the liver and kidney, respectively. In the canonical mechanism of action of vitamin D, calcitriol acts as the ligand for the vitamin D receptor (VDR), acting in conjunction with the retinoid X receptor (RXR). This complex modulates gene expression by binding to vitamin D response elements (VDREs) located in the promoters of target genes [5]. This transcriptional regulation, mediated by the VDR-RXR heterodimer, underscores the role of calcitriol in many biological processes, including its anti-tumoral effects, such as inhibiting proliferation, migration, metastasis, angiogenesis, and promoting differentiation and apoptosis [6].

Several studies have explored the effects of calcitriol on the transcriptome of both normal and tumor cells using methodologies like microarrays or RNA-seq [7–10]. However, the transcriptional regulatory mechanisms driven by calcitriol in CaSki cervical cancer cells, which harbor integrated high-copy numbers of HPV16, remain unexplored. This study sought to address this gap through microarray analysis. Key findings from the microarray were validated using qPCR and western blot analyses, and the impact of calcitriol on CaSki cell proliferation and migration was also assessed.

## Materials and methods

### Cell culture

The human cervical cancer cell line CaSki, obtained from the American Type Culture Collection (CRL-1550, Manassas, VA, USA), was cultured in growth medium (RPMI 1640 medium supplemented with 5% heat-inactivated fetal bovine serum, 100 units/mL penicillin, and 100 µg/mL streptomycin). For experimental treatments, cells were seeded and incubated for 24 hours before being exposed to either calcitriol at a concentration of 10 nM (Sigma-Aldrich, St. Louis, MO, USA) or 0.1% ethanol as a vehicle for an additional 24 hours period in treatment medium (RPMI medium supplemented with 5% charcoal-stripped fetal bovine serum).

### Immunofluorescence analysis

CaSki cells were seeded (150,000 cells) onto 4-well cell culture slides in growth medium. After 24 hours, the medium was aspirated and replaced with either 0.1% ethanol as vehicle or 10 nM calcitriol in treatment medium. Cells were then incubated for 24 hours, washed twice with warm saline solution, and fixed with absolute ethanol at -20°C for 8 minutes. Following fixation, cells were permeabilized and blocked in fixation/permeabilization solution for 1 hour at room temperature (BD Biosciences, Franklin Lakes, NJ, USA). Slides were incubated overnight at 4°C with primary antibodies against VDR (mouse monoclonal anti-VDR antibody, 1:100, sc-13133X, Santa Cruz Biotechnology, Dallas, TX, USA) and RXR (rabbit polyclonal anti RXR α,β,γ antibody, 1:100, sc-774X, Santa Cruz Biotechnology). Negative controls were incubated with perm/wash solution (BD Biosciences) without primary antibodies. After primary antibody incubation and 3 washes, slides were incubated for 1 hour at room temperature with goat anti-mouse IgG secondary antibody conjugated with Cy3 (1:1000, A10521, Life Technologies, Carlsbad, CA, USA) and goat anti-rabbit IgG secondary antibody conjugated with Alexa Fluor 488 (1:500, 111-547-003, Jackson ImmunoResearch, West Grove, PA, USA). Slides were cover-slipped with mounting medium containing DAPI (UltraCruz Hard-set Mounting Medium, Santa Cruz Biotechnology) and analyzed using an epifluorescence microscope (Olympus BX51, Japan).

### Cell proliferation studies

To study the effect of calcitriol on the proliferation of CaSki cells, 2000 cells per well were seeded in a 96-well plate. Following a 24-hour incubation period to allow for cell adhesion, either 0.1% ethanol as a vehicle or 10 nM calcitriol in the treatment medium were added. The cells were then incubated for 1, 2, 3 or 4 days, with daily replenishment of each treatment. Cell proliferation was subsequently determined using the XTT Cell Proliferation Kit II (Roche, Germany) following the manufacturer’s instructions.

### Wound healing assay

In the interest of validating the effects of calcitriol upon cell migration pathways, we evaluated whether the migration capacity of CaSki cells could be regulated by calcitriol utilizing the wound healing assay. For this, CaSki cells were seeded in 24-well plates (500,000 cells/well) until confluence, after which, a cross-shaped scratch was created in the cell monolayer using a sterile pipette tip. The cells were then washed to remove any detached cells and debris. Afterwards, the cells were incubated with 5 μM cytosine arabinoside for 48 h in the presence of vehicle (ethanol 0.1%) or 10 nM calcitriol. The cells were photographed at time 0 and after 48 h to evaluate the wound area at the beginning and at the end of treatment. Measurements were conducted using ImageJ software, analyzing four different wells for each experimental condition. Comparisons between vehicle and calcitriol treatments were evaluated by Student’s t-test using SigmaPlot software.

### RNA isolation

Total RNA extraction was performed using the standard protocol described by Chomczynski and Sacchi employing Trizol reagent [11]. The obtained RNA was quantified spectrophotometrically, and the integrity of the RNA samples was assessed using an Agilent 2100 Bioanalyzer (Agilent Technologies, Santa Clara, CA, USA), ensuring that the RNA samples exhibited an integrity value equal to or greater than 9.0 to undergo processing for microarray analysis.

### Reverse transcription/real-time PCR (qPCR)

The cDNA synthesis was conducted utilizing 5 µg of total RNA as a template and the Maxima First Strand cDNA Synthesis Kit (ThermoScientific, Waltham, MA, USA). Subsequently, qPCR experiments were performed using TaqMan Master reagents, specific hydrolysis probes from the Human Universal Probe Library (UPL, Roche, Germany) Set, and a LightCycler 480 II Instrument from Roche. The oligonucleotide sequences (5’-3’, forward/reverse) and corresponding UPL numbers are indicated in Table 1.

**Table 1:** Oligonucleotide sequences and hydrolysis probes used in this study.

Gene ID	Accession number	Sequence 5’-3’ (forward/reverse)	UPL number
*CYP24A1*	NM_000782	CATCATGGCCATCAAAACAA GCAGCTCGACTGGAGTGAC	88
*KLK6*	NM_002774	TGCTGAGTCTGATTGCTGCA TGAGATGTCTTGTCGCAGGG	68
*CYP4F3*	NM_000896	AAAGCACCCGGAATACCAGG TAGGCTCACGGTCCTTCAGA	51
*RARA*	NM_000964	GAATCCTGAATCGAGCTGAGA GGGCCATGTCCTGTGATG	2
*EPHA2*	NM_004431	CAGCTCATGATGCAGTGCTG AGCTTGTCCAGGATGCTGAC	70
*MMP7*	NM_002423.3	GCTGACATCATGATTGGCTTT TCTCCTCCGAGACCTGTCC	72
*MMP13*	NM_002427.3	CCAGTCTCCGAGGAGAAACA AAAAACAGCTCCGCATCAAC	73
*RPL32*	NM_000994	GAAGTTCCTGGTCCACAACG GAGCGATCTCGGCACAGTA	11

The PCR protocol involved an initial denaturation step at 95°C for 10 min, followed by 45 cycles at 94°C for 10 s, 60°C for 30 s, and 72°C for 1 s. Ribosomal protein L32 (RPL32) expression was an internal control for normalizing gene expression data using the LightCycler Software 4.05 (Roche, Germany).

### Western blot analysis

Cell lysis was conducted using RIPA buffer (9.1 mM dibasic sodium phosphate, 1.7 mM monobasic sodium phosphate, 150 mM NaCl, 1% Nonidet P-40, 0.1% SDS, pH 7.4), supplemented with protease inhibitors (Complete, Roche, Germany). Protein concentration was determined using the bicinchoninic acid method (Thermo Scientific) [12]. Subsequently, proteins were separated by electrophoresis on 12% denaturing polyacrylamide gels, and then transfered onto nitrocellulose membranes by the semi-dry method. The membranes were blocked using a 5% skim milk solution in TBS buffer (10 mM Tris-HCl, 50 mM NaCl; pH 8.0). Membranes were initially incubated with primary antibodies (dilution 1:300) targeting EPHA2 (sc-398832), RARΑ (sc-100907), KLK6 (sc-374564), and CYP4F3 (sc-515735), obtained from Santa Cruz Biotechnology. Subsequently, appropriate Santa Cruz Biotechnology secondary antibodies were used. Visualization of the target proteins was achieved using a chemiluminescence detection system (Pierce ECL Western Blotting Substrate, Thermo Scientific) in conjunction with a Molecular Imager ChemiDoc XRS System and Image Lab Software (Bio-Rad, Hercules, CA, USA). For loading control, an anti-GAPDH antibody (1:300, sc-365062, Santa Cruz Biotechnology), was used.

### Microarray and pathway analysis

For this study, three independent experiments were conducted, each comprising three experimental replicates per experimental condition. From each experimental condition, 3 pools of total RNA were prepared, with each pool comprising an equimolar combination of total RNA from three experimental replicates across three different experiments. Subsequently, each of the six pools was subjected to analysis using Clariom D human microarrays (Affymetrix, Santa Clara, CA, USA) to assess global gene expression profiles. The hybridization, washing, and scanning procedures were performed following the manufacturer’s protocol. Microarray data were submitted to Gene Expression Omnibus (GEO) with accession number GSE267715.

Background correction, normalization, and final analysis of the differentially expressed profiles were performed using Transcriptome Analysis Console (TAC) Software v4.0.1 (Thermo Fisher Scientific). Genes with a log fold change of ± 1.5 and a false discovery rate < 0.05 were considered statistically significant. Furthermore, a comprehensive pathway enrichment analysis was conducted using the Ingenuity Pathway Analysis tool (version 111725566, Qiagen, Germany).

### Statistical analysis

Statistical differences were evaluated using one-way ANOVA followed by post hoc tests employing the Holm-Sidak method for pairwise comparisons. This analysis used specialized software (SigmaPlot 12.0, Systat Software, Inc. Chicago, IL, USA). Statistical significance was set at a threshold of *P* <  0.05.

## Results

### Characterization of calcitriol-related receptors in CaSki cell line

**Fig 1:**
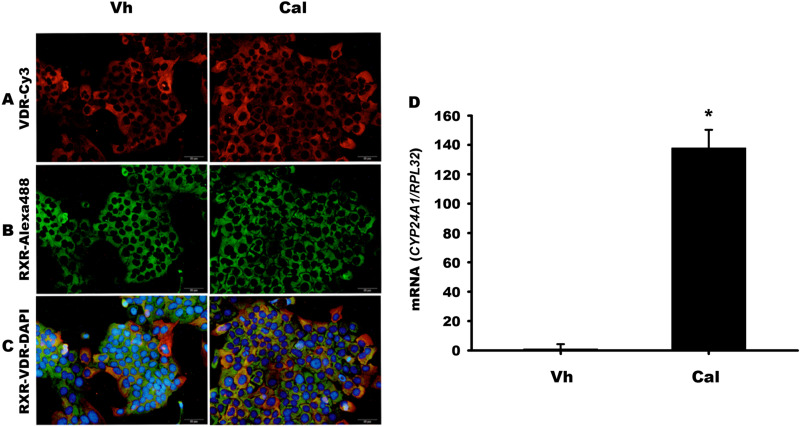
CaSki cells functionally express VDR and RXR. (A) 150,000 CaSki cells were seeded onto 4-well chamber slides and after 24 hours, they were further incubated for 24 hours with either 0.1% ethanol as a vehicle (Vh) or 10 nM calcitriol (Cal). Protein expression was evaluated by immunofluorescence. VDR is shown in red (A), RXR α, β, and γ subtypes in green (B), and merged in (C). DAPI-stained nuclei appear in blue. Magnification 20X. (D) Calcitriol significantly induces *CYP24A1* gene expression in CaSki cells. Cells were cultured for 24 hours and subsequently incubated with either Vh or Cal. After 24 hours, total RNA was extracted, and *CYP24A1* gene expression was evaluated by qPCR. Bars represent the mean of 3 independent experiments (each with at least 3 replicates) ±  SD. * *P* < 0.05 vs. Vh.

First, we investigated whether CaSki cells could be a suitable model for evaluating calcitriol-mediated effects. To this end, we examined whether CaSki cells express VDR and RXR by immunofluorescence. As depicted in Fig 1A, Fig 1B, and, Fig 1C, both receptors are abundantly expressed in the cytoplasm of CaSki cells but were not observed in the nuclei. At the time frame tested, treatment of CaSki cells with calcitriol did not alter the expression or distribution of either VDR or RXR. To assess the functionality of these receptors following calcitriol stimulation, we examined the expression of the *CYP24A1* gene, encoding the catabolic enzyme of calcitriol, 25-hydroxyvitamin D_3_ 24-hydroxylase, which is recognized as the most responsive gene to calcitriol stimulation [13]. As shown in Fig 1D, *CYP24A1* mRNA increased over 100-fold following calcitriol stimulation, demonstrating the functionality of VDR and RXR in CaSki cells. These findings demonstrate the presence of functional VDR and RXR receptors and validate CaSki cells as a suitable model for investigating calcitriol-mediated mechanisms in cervical cancer.

### Calcitriol represses CaSki cell transcriptome

**Fig 2:**
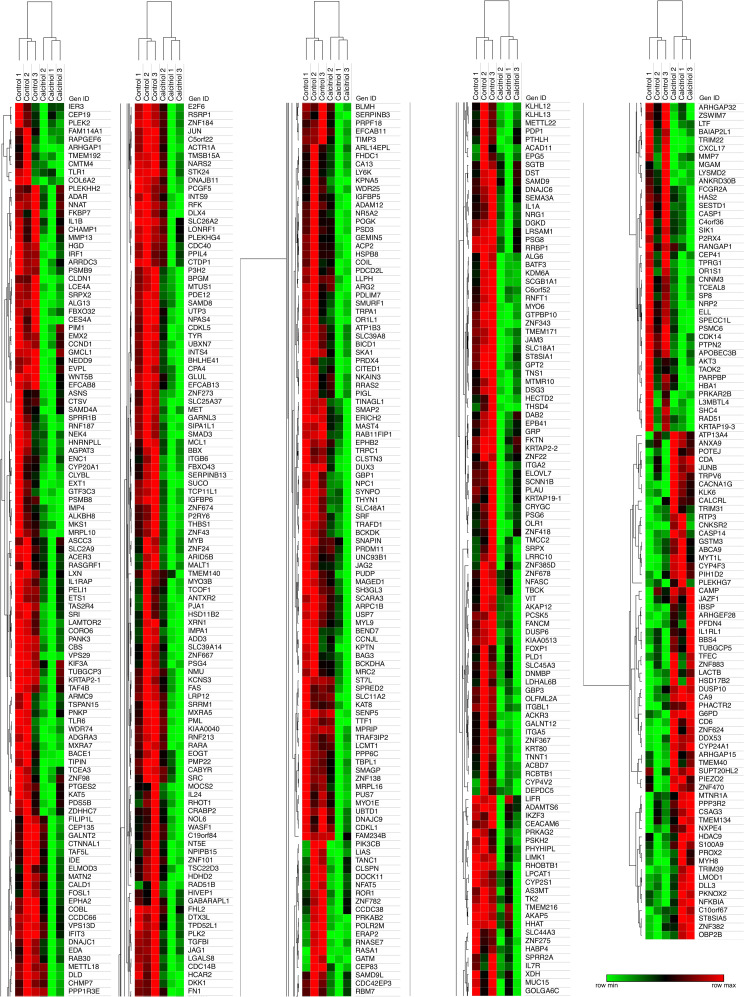
Heatmaps of the top 536 differentially expressed genes (DEG) by calcitriol in CaSki cells with fold changes ranging from 1.5 to −1.5. To display all DEG, a single heatmap obtained was split into 5 parts. Five columns are presented, of which 4 correspond to genes repressed by calcitriol, while the last column shows the genes induced by calcitriol. The green color denotes lower expression levels, while red indicates higher expression levels.

Next, we decided to investigate the global effect of calcitriol on the transcriptome of CaSki cells using microarray analysis. In exploring the transcriptomic landscape following calcitriol treatment in CaSki cells, our analysis revealed a set of genes displaying differential expression patterns between the calcitriol-treated and control groups (S1 Table). Specifically, our hierarchical clustering analysis (Fig 2) reveals that calcitriol treatment resulted in the downregulation of 474 coding genes (88%) and the upregulation of 62 coding genes (12%), including the previously validated *CYP24A1* transcript (Fig 1D). Notably, while the alterations in expression reach statistical significance, most of the genes depicted in Fig 2 demonstrate modest fold change values ranging between 1.5 and 2.

Microarray analysis revealed that calcitriol not only regulates protein-coding genes but also significantly alters the expression of approximately 400 non-coding RNAs (S1 Table). Among these, calcitriol modulated a total of 111 microRNA precursors, inducing 69 and repressing 42. Furthermore, calcitriol exhibited regulatory effects on mature microRNAs, upregulating 17 and downregulating 12 (Table 2). Notably, several of these calcitriol-regulated microRNAs, including miR-6129, miR-382, miR-655, miR-211, miR-590, miR-130a, miR-301a, and miR-1252, are implicated in cervical cancer biology.

**Table 2:** Differentially expressed mature microRNAs in CaSki cells modulated by calcitriol, with fold change thresholds of ≥ 1.5 or ≤ -1.5.

MicroRNA induced by calcitriol	Fold change	MicroRNA suppressed by calcitriol	Fold change
miR-3646	3.16	miR-4421	−1.54
miR-548ae-1	2.41	miR-579	−1.56
miR-3128	2.21	miR-649	−1.58
miR-6129	1.94	miR-4503	−1.63
miR-3167	1.74	miR-130a	−1.71
miR-6854	1.72	miR-4637	−1.73
miR-5007	1.72	miR-4477a	−1.81
miR-382	1.71	miR-7111	−1.85
miR-4705	1.67	miR-301a	−1.87
miR-655	1.63	miR-548ap	−2.16
miR-4652	1.62	miR-1252	−2.35
miR-211	1.62	miR-5692c-2	−2.45
miR-606	1.60		
miR-590	1.58		
miR-6817	1.55		
miR-5688	1.53		
miR-4499	1.51		

**Fig 3:**
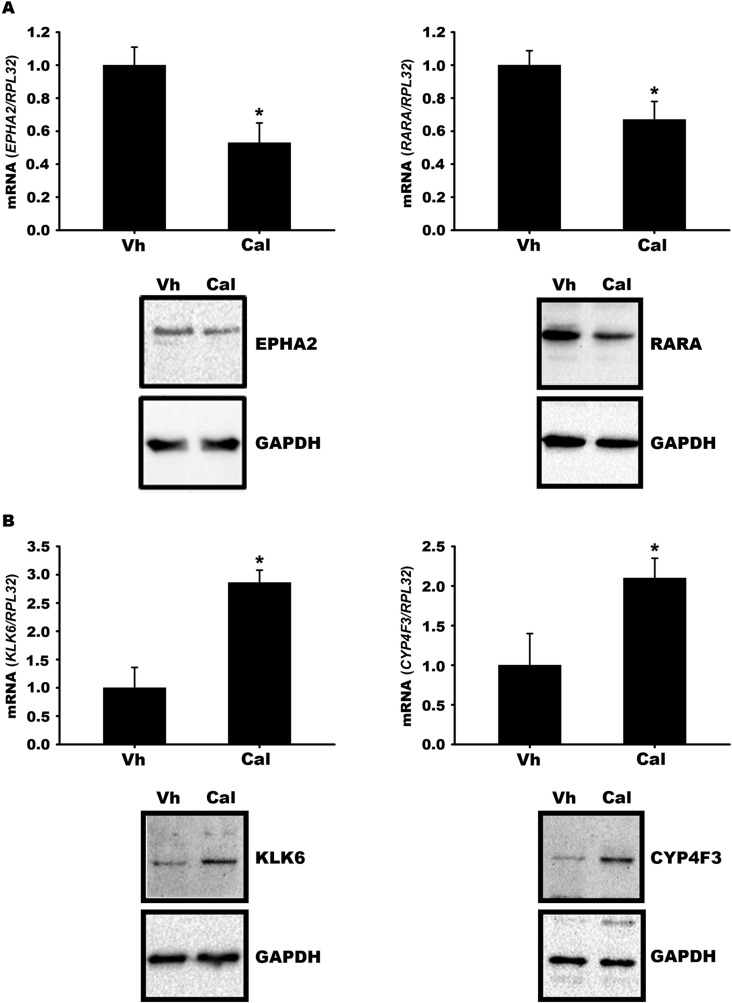
Validation of gene expression changes induced by calcitriol in CaSki cells. Calcitriol downregulates the expression of *EPHA2* and *RARA* (A) while inducing *KLK6* and *CYP4F3* (B) at mRNA and protein levels. CaSki cells were cultured for 24 hours and treated with either vehicle (Vh) or calcitriol (Cal). After incubation, RNA was extracted and the expression of the target genes were assessed by qPCR. Bars represent the mean of 3 independent experiments (each with at least 3 replicates) ±  SD. * *P* < 0.05 vs. Vh. Protein abundance was assessed through Western blot analysis employing suitable antibodies and representative western blot membranes for each of the proteins studied are shown.

We selected some differentially expressed genes (DEG) by calcitriol in CaSki cells for validation by qPCR and western blot analysis. EPH Receptor A2 (*EPHA2*) and retinoic acid receptor alpha (*RARA*) genes, which were repressed by calcitriol in the microarray, were also suppressed by calcitriol at both mRNA and protein levels (Fig 3A). Conversely, the transcripts and protein abundance of kallirrein related peptidase 6 (*KLK6*) and cytochrome P450 family 4 subfamily F member 3 (*CYP4F3*) were induced by calcitriol (Fig 3B), similar to what was observed in the microarray. These results validate the transcriptomic changes exerted by calcitriol in CaSki cervical cancer cells.

### Biological impact of differentially expressed genes

**Fig 4:**
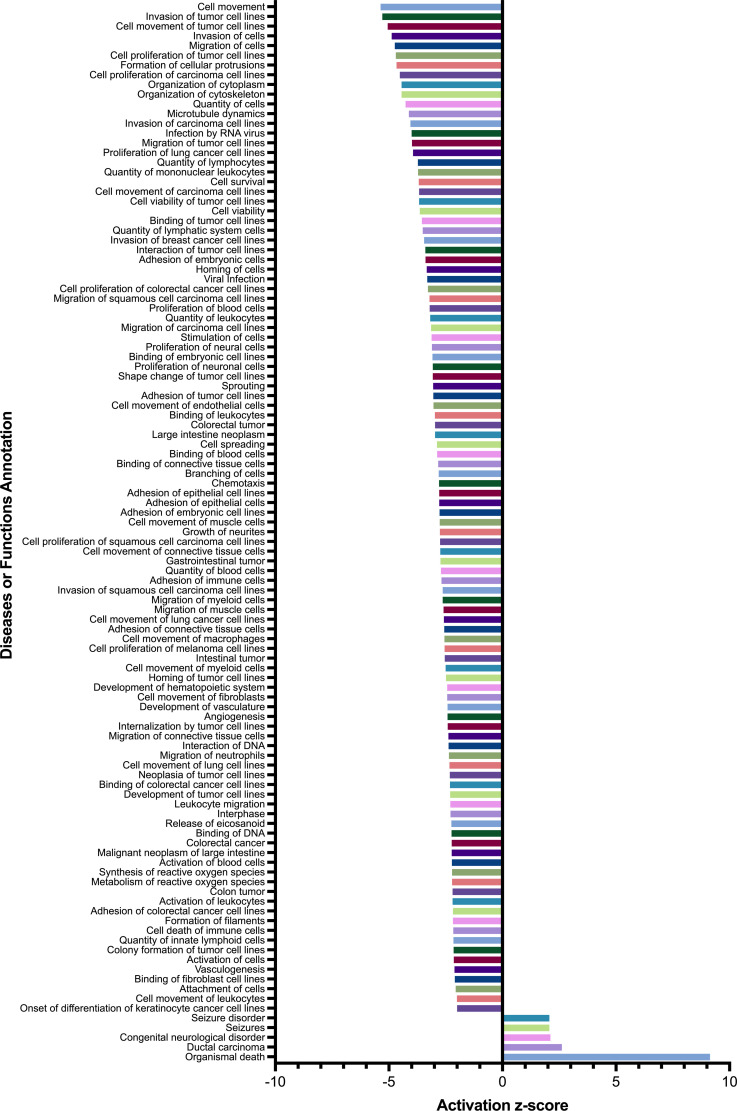
Biological impact of differentially expressed genes by calcitriol in CaSki cervical cancer cells. Diseases and functions obtained from differential expression profiling were analyzed using Ingenuity Pathway Analysis, showcasing the most significant findings. The z-score refers to the value for the predicted activation state of the biological process.

We conducted a pathway enrichment analysis to ascertain the biological implications of these transcriptional changes. The findings revealed that calcitriol predominantly suppresses several pro-carcinogenic biological processes such as cell proliferation, movement and spreading, invasion, adhesion, migration, angiogenesis, and vasculogenesis (Fig 4). Notably, the organismal death pathway, which generally includes apoptosis, necroptosis, pyroptosis, and ferroptosis, was positively regulated, exhibiting the highest z-score among the analyzed processes (Fig 4). A detailed list of both activated and suppressed biological processes, along with the genes associated with these pathways, is provided in S2 Table. Additionally, the analysis highlighted the negative regulation by calcitriol of genes associated with specific immunological processes, including the adhesion and cell death of immune cells. This regulatory effect on immune-related genes further emphasizes the comprehensive role of calcitriol in modulating the immune environment within the tumor microenvironment. Interestingly, pathway enrichment analysis reveals that calcitriol demonstrates suppressive effects against viral infections

### Predicted interaction network of calcitriol-dependent differentially expressed genes in CaSki cells

**Fig 5:**
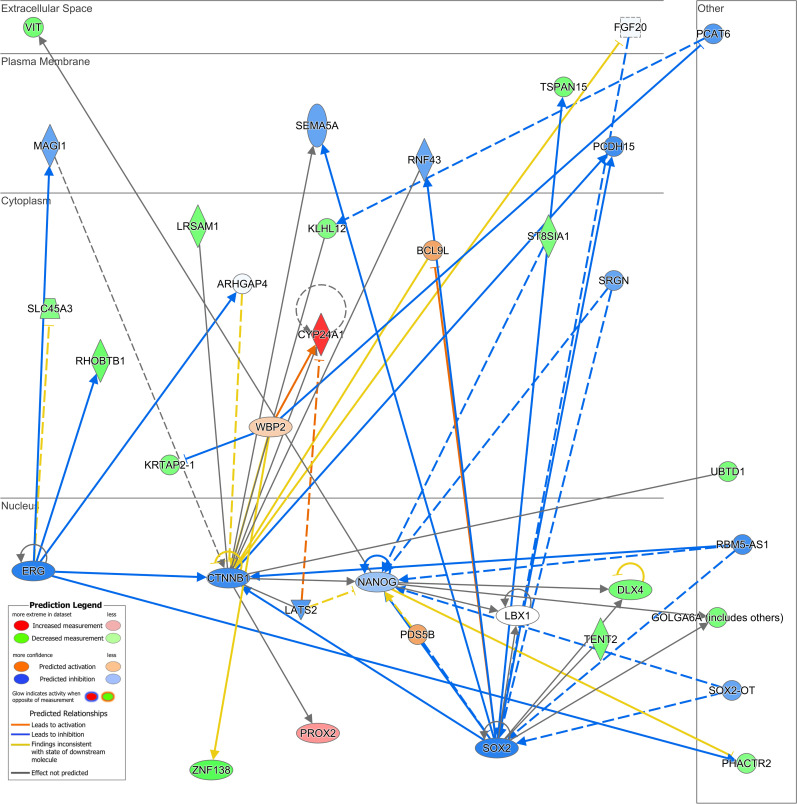
Predicted interaction network of differentially expressed genes by calcitriol in CaSki cervical cancer cells. The panel shows the legend for the predictions and interactions among the genes constructed using Ingenuity Pathway Analysis. The lines connecting the labels represent physical or functional interactions between the proteins. Solid lines represent known direct interactions, while dashed lines represent predicted or indirect interactions.

Further analysis focused on the predicted interaction network of the DEG, providing insights into their potential roles within cellular compartments. Fig 5 displays the predicted interaction network among DEG, highlighting significant nodes such as transcription factors and signaling pathway components. Notably, the mRNA levels of these predicted nodes remain unchanged following calcitriol treatment. The network is segmented by cellular compartments, emphasizing the multifaceted impact of calcitriol on cellular functions. Key transcription factors like SOX2 and NANOG, known for their crucial roles in gene regulation and stem cell maintenance, were among those prominently interacting within the network.

ERG, another transcription factor with critical functions in cell proliferation and differentiation, was also evident in the predicted interaction network. Moreover, the network analysis underscored significant interactions with CTNNB1, encoding catenin beta and involved in coordinating cell-cell adhesion and gene transcription. Finally, another notable protein in the interaction network is PCDH15, a cadherin superfamily member of calcium-dependent transmembrane cell adhesion proteins.

### Calcitriol suppresses CaSki cell proliferation

**Fig 6:**
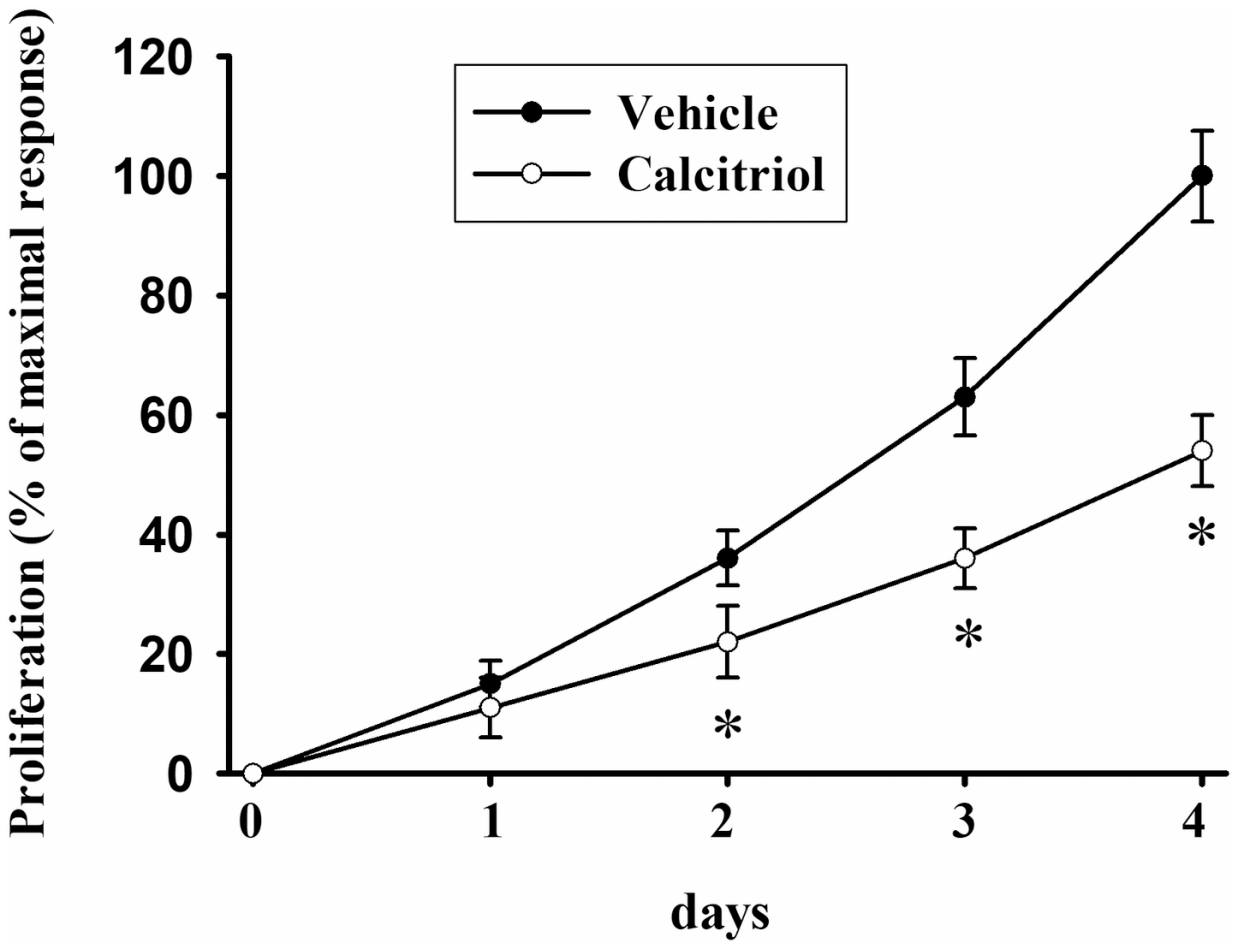
Calcitriol suppresses proliferation of CaSki cells. Cells were treated daily with either vehicle or 10 nM calcitriol at the specified times in treatment medium. Day 0 corresponds to the time when treatments were added, 24 hours after plating the cells. Cell growth was assessed daily using XTT assay. Each data point represents the average of 3 independent experiments, each with three experimental replicates ±  the SD of proliferation normalized against data of vehicle-treated cells at day 4, which was considered as 100%. * *P* < 0.05 vs. Vehicle.

The results of Fig 4 strongly suggest that calcitriol suppresses key processes relevant to tumor progression, such as cell proliferation and migration. As expected, Fig 6 demonstrates that calcitriol significantly inhibited the proliferation of CaSki cells starting from the second day of incubation.

### Calcitriol suppresses CaSki cell migration

**Fig 7:**
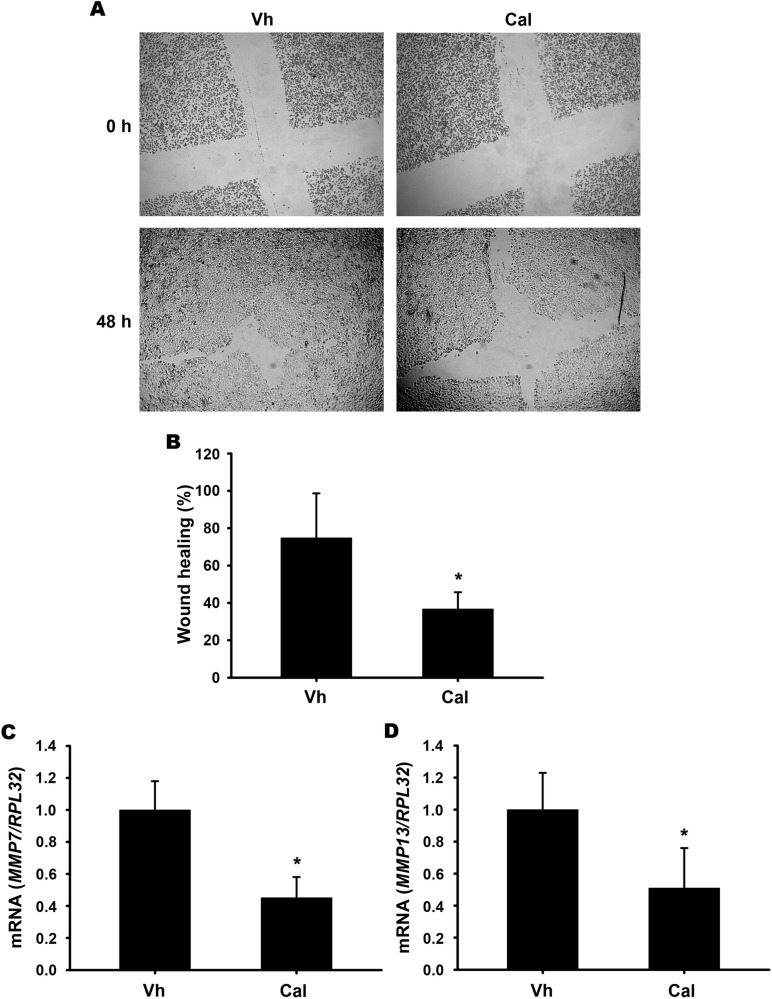
Calcitriol inhibits CaSki cell migration and suppresses MMP7 and MMP13 gene expression. The wound healing assay demonstrated that calcitriol delayed CaSki cell migration (A). Cells were seeded in 24-well plates, and a cross-shaped scratch was made. They were treated with vehicle (Vh) or 10 nM calcitriol (Cal) for 48 hours. Bright-field images (4×) were taken at 0 and 48 hours to assess the wound area. (B) Quantitative analysis of wound healing revealed significant suppression of migration. For gene expression studies, CaSki cells were cultured for 24 hours and treated with either vehicle (Vh) or 10 nM calcitriol (Cal). Total RNA was extracted, and the expression levels of MMP7 (C) and MMP13 (D) genes, along with the housekeeping gene RPL32, were quantified using qPCR. Data are presented as the mean ±  SD of three independent experiments. * *P* < 0.05 vs. Vh.

The wound healing assay of CaSki cells was conducted to validate the effect of calcitriol on cell migration, as suggested by the results in Fig 4. As shown in Fig 7A and 7B, calcitriol significantly reduced the migratory capacity of CaSki cells after 48 hours of treatment. This observation was further supported by the inhibition of MMP7 and MMP13 gene expression (Fig 7C and 7D). Collectively, these results confirm that calcitriol acts as an effective suppressor of CaSki cell migration.

## Discussion

An essential requirement for vitamin D responsiveness is cellular expression of VDR, which is widely distributed in the human body, including female reproductive tissues [4,14]. Both normal and tumoral cervical tissues harbor the enzymatic machinery for calcitriol biosynthesis, suggesting local non-calcemic hormonal actions [15,16]. Notably, established cervical cell lines, such as HeLa [17] and SiHa [18], express VDR, while C-33A cells lack it [19]. It has been previously described that CaSki cells express low basal levels of RXR receptors [20]. Our study reveals abundant VDR and RXR expression in CaSki cells, primarily in the cytoplasm. High cytoplasmic VDR staining correlates with poor overall survival in ovarian cancer [21] and is associated with tumor progression and prognosis in colon and vulvar cancers [22,23], implying the involvement of non-nuclear VDR-mediated signaling pathways in these tumors [24]. While the specific role of non-genomic VDR effects in CaSki cells is yet to be elucidated, our results from the genomic-mediated induction of calcitriol on the *CYP24A1* gene and comprehensive transcriptome microarray data indicate that activated VDR predominantly functions through the classical nuclear pathway, modulating gene expression via promoters containing VDREs.

Regarding the effect of calcitriol on the transcriptome of CaSki cells, we observed numerous genes subjected to hormonal regulation using a fold change criterion of ±  1.5. Numerous studies have explored the transcriptional regulation mediated by calcitriol or its analogs across various cancer types, such as colon, prostate, and breast cancer, using microarray or RNA-seq techniques [7–9]. These investigations have consistently shown that calcitriol typically upregulates gene expression in colon, prostate, and breast cancer cells [7,8,25,26]. Remarkably, our study revealed an 88% repression in the transcriptome of CaSki cells following calcitriol treatment. To our knowledge, no previous studies have reported such a substantial repressive effect of calcitriol on the transcriptome. A study on chronic lymphocytic leukemia cells also reported transcriptome repression, albeit to a lesser extent, with genes being repressed twice as frequently as induced [10], aligning with the anti-inflammatory role of calcitriol in immune cells [27].

Among the diverse genes affected by calcitriol, primarily protein-coding genes were identified, along with long non-coding RNAs and various microRNAs. MicroRNAs regulate target genes at the post-transcriptional level mainly by inducing their mRNA degradation and repressing their mRNA translation. Interestingly, we observed that the number of genes repressed by calcitriol (474) exceeded those induced by calcitriol (62) by more than sevenfold, indicating a potent suppressive influence of calcitriol on the CaSki cell transcriptome. In this study, several microRNAs known for their capacity to inhibit transcription exhibited a calcitriol-dependent upregulation, further supporting a strong transcription repressive activity of calcitriol in CaSki cervical cancer cells. However, while in cervical cancer the downregulation of microRNAs has been mostly associated with a poor prognosis [28], further analysis are needed to asseverate the biological outcome of calcitriol through the regulation of the microRNAs found in this study.

Our microarray analysis demonstrated that, beyond its influence on protein-coding genes, calcitriol significantly modulates non-coding RNA expression, with 55% of non-coding RNAs upregulated and 45% downregulated. Although changes in microRNA expression were not experimentally validated, the data suggest that calcitriol upregulated 62% of mature microRNAs and downregulated 38%, potentially contributing to its mRNA-suppressive effects. Among the microRNAs whose expression was modulated by calcitriol, only a few have been previously identified as calcitriol targets. Our group previously reported the microRNA changes induced by calcitriol treatment in SiHa cervical cancer cells [29]. Notably, the microRNA alterations observed in SiHa cells differ markedly from those identified in CaSki cells in this study. This observation is consistent with previous findings in cervical cancer, where a distinct microRNA expression profile has not been conclusively established for this cancer type or specific HPV strains [30]. Interestingly, calcitriol consistently inhibited miR-548 expression and increased miR-590 expression in both cell types. However, notable differences were observed: miR-3128 and miR-606 were significantly upregulated by calcitriol in CaSki cells but were repressed in SiHa cells [29].

The majority of microRNAs upregulated by calcitriol in this study are known to play significant roles in cancer pathogenesis, with a subset specifically implicated in cervical carcinogenesis. Notably, calcitriol increased the expression of several microRNAs in CaSki cells, including miR-6129, miR-382, miR-655, miR-211, and miR-590. Interestingly, these calcitriol-regulated microRNAs may exert antitumoral activities in CaSki cervical cancer. Specifically, miR-6129 has been suggested to disrupt cellular metabolic reprogramming by promoting the degradation of HIF-1α, a protein whose expression is influenced by HPV E6 and E7. This anticancer mechanism of calcitriol is thought to be indirect, as cullin 2, a key factor required for HIF-1α degradation, is a target of miR-6129 [31]. Conversely, miR-382-5p is downregulated in cervical cancer, where its predicted target, VEGFA, is highly expressed, promoting tumor growth and metastasis. In this study, calcitriol upregulated miR-382-5p in CaSki cells, potentially reducing VEGFA expression and its tumor-promoting activity [32]. Interestingly, calcitriol also increased the expression of miR-655, a microRNA previously reported to be downregulated in HPV16-positive cervical cancer [33]. Another calcitriol-induced microRNA, miR-211, has been shown to trigger autophagy and autophagy-dependent apoptosis through the regulation of Bcl-2 in cervical cancer cells [34]. Notably, calcitriol upregulated miR-590 expression in CaSki cells. Among the microRNAs induced by calcitriol, miR-590 stands out as the only one with evidence of promoting cervical cancer cell growth and invasion by targeting CHL1 [35]. This finding merits further exploration in future research.

Conversely, among the microRNAs downregulated by calcitriol in CaSki cells are miR-130a, miR-301a, and miR-1252. MiR-130a has previously been shown to enhance its antiviral effects on hepatitis C virus replication when potentiated by calcitriol, independent of type I interferon signaling [36]. This microRNA also plays a significant role in the pathogenesis of cervical cancer by targeting Dicer [37], thereby promoting cell migration and invasion in SiHa cells. Therefore, further investigation into the role of microRNA-130a in CaSki cells is needed. MiR-301a is another microRNA repressed by calcitriol. It has been reported to be significantly upregulated in cervical carcinoma tissues, while its target, phosphatase and tensin homolog (PTEN), was notably downregulated. This dysregulation is associated with enhanced cancer cell proliferation and reduced apoptosis [38]. Finally, calcitriol downregulated miR-1252, which targets EIF4EBP2, a factor involved in proto-oncogene regulation. By repressing miR-1252, calcitriol may help reduce cervical cancer progression [39]. Collectively, these findings suggest that calcitriol contributes to the suppression of cervical cancer progression by downregulating key microRNAs involved in tumorigenic processes.

Besides serving as a model for advanced cervical cancer due to its high HPV16 viral load and numerous cellular alterations [40,41], CaSki cells demonstrate significant sensitivity to external factors. This is exemplified by the ability of α-mangostin, a plant derived antineoplasic compound, to inhibit CaSki cell proliferation by suppressing HPV16 E6/E7 and *KCNH1* oncogenes [42]. This increased vulnerability of CaSki cells might explain the profound transcriptional effects induced by calcitriol. Interestingly, the biological significance of these transcriptomic changes induced by calcitriol also demonstrated widespread repression across virtually all analyzed categories. Among the prominent biological processes repressed by calcitriol, involving more than 100 molecules, are cell movement, cell survival, cytoplasm organization, cell proliferation, and cell quantity.

Uncontrolled proliferation, a hallmark of cancer, is driven by genetic mutations, disrupted cell cycle checkpoints, and tumor microenvironments like hypoxia, leading to tumor growth, metastasis, and therapy resistance [43]. Cell proliferation represents a pivotal biological process that undergoes negative regulation by calcitriol across various cell types [44]. In our study, calcitriol significantly inhibited CaSki cell proliferation compared to the vehicle, starting from two days post-hormone addition, maintaining a 50% suppression until day 4. Although there are no studies on the effect of calcitriol on CaSki cell proliferation, it has been previously reported that high concentrations of vitamin D_3_ significantly inhibit CaSki cell proliferation and activate apoptosis [45]. This effect is likely mediated by the endogenous conversion of vitamin D_3_ to calcitriol within CaSki cells, leading to an increase in the sub-G_1_ cell cycle fraction indicative of cellular death. Consistent with other studies on calcitriol or its precursor 25-hydroxyvitamin D_3_, these vitamin D_3_ derivatives induce time- and concentration-dependent suppression of cell proliferation and G_1_ cell cycle arrest in cervical cancer cells such as HeLa and SiHa [46–48]. These findings, along with our results, highlight the antiproliferative potential of vitamin D derivatives in cervical cancer cells [49]. In general, calcitriol inhibits tumor cell proliferation through multiple mechanisms, including inducing cell cycle arrest by downregulating c-Myc and cyclins, suppressing the Wnt/β-catenin pathway to reduce glycolysis, and promoting differentiation for less aggressive tumor phenotypes. Calcitriol reduces tumor volume and enhances chemotherapy efficacy in animal models, highlighting its potential as a complementary cancer therapy [6].

In addition to proliferation, cancer cell migration plays a crucial role in tumor progression and metastasis, facilitating the invasion of surrounding tissues and the spread to distant sites [50]. This process is influenced by metabolic reprogramming, such as the Warburg effect, genetic alterations like epithelial-to-mesenchymal transition (EMT), and biomechanical interactions with the extracellular matrix (ECM). Specific proteins, such as MIEN1, further enhance migration, collectively contributing to tumor invasiveness.

Considering the predicted biological processes influenced by calcitriol treatment in CaSki cells, it was evident that processes related to cell movement, including migration, were significantly inhibited. This study demonstrated that calcitriol significantly reduced the migratory capacity of CaSki cells after 48 hours in the wound healing assay. This effect was further supported by the downregulation of *MMP7* and *MMP13* gene expression, both critical molecules involved in cell migration [51]. Our results align with other studies suggesting that calcitriol generally inhibits tumor cell migration [52,53]. Calcitriol acts by suppressing the expression of several metalloproteinases and modulating cytokine production, thereby reducing inflammation and tumor invasiveness. Tumor cells promote invasion by upregulating matrix metalloproteinases (MMPs), including MMP7 and MMP13 [51].

MMP7 promotes cancer progression by degrading the extracellular matrix (ECM), enhancing epithelial-mesenchymal transition (EMT), supporting angiogenesis, and modulating the immune response, all of which contribute to increased metastasis [54]. This study found that calcitriol significantly suppressed MMP7 mRNA expression in CaSki cervical cancer cells, an effect also observed in other cancer types [55]. While protein level data were not provided, calcitriol likely downregulates MMP7 activity, helping reduce cell migration. Elevated MMP7 levels correlate with higher cancer grade, clinical stage, and metastasis in cervical cancer, suggesting its potential as a clinical biomarker for the disease [56]. Additionally, calcitriol significantly suppressed MMP13 mRNA expression in CaSki cervical cancer cells, consistent with previous studies in squamous cell carcinoma and breast cancer cells treated with the synthetic vitamin D derivative calcipotriol and the low-calcemic vitamin D analog MART-10, respectively [57,58]. Similar to MMP7, MMP13 is a key enzyme in cancer progression, promoting tumor cell migration, metastasis, and angiogenesis by degrading the extracellular matrix and activating epithelial-mesenchymal transition (EMT), thereby enhancing tumor invasiveness [59]. Notably, higher levels of MMP13 were observed in cervical cancer tissues compared to adjacent noncancerous tissues, with these elevated levels correlating with a poorer prognosis for cervical cancer patients [60]. Thus, it seems that part of the beneficial effects of calcitriol on cervical cancer may be attributed to its suppression of MMP7 and MMP-13 gene expression.

During the validation phase of our microarray experiments, we targeted specific genes based on their potential relevance to cancer biology. Specifically, we noted that calcitriol downregulated the *EPHA2* and *RARA* genes in the microarray data. This observation was subsequently confirmed at the mRNA and protein levels in CaSki cells. EPHA2 belongs to the ephrin-A receptor subfamily of the tyrosine kinase protein family and has been shown to have oncogenic functions in various cancer types [61], including cervical cancer [62,63]. Retinoic acid receptor α is overexpressed in breast cancer tumors [64] and other neoplasias [65], contributing to tumoral progression. Little is known about the role of RARΑ in cervical cancer. Available evidence indicates the expression of the RARA gene in HeLa and CaSki cells [66]. Additionally, RARA expression is reduced by 75% in cervical tumors compared to control tissues [67]. Therefore, further studies are needed to understand the effect of calcitriol-induced RARA reduction in CaSki cells.

Moreover, among the genes upregulated by calcitriol in the microarray analysis are *KLK6* (kallikrein-related peptidase 6) and *CYP4F3* (cytochrome P450 family 4 subfamily F member 3). The serine protease KLK6, participates in diverse cancer-associated processes [68]. This gene can have oncogenic or antitumoral effects depending on the tumor. In various tumors, KLK6 is abundantly expressed [68,69], while in others, its expression is deficient. In breast cancer, *KLK6* acts as a tumor suppressor gene but its expression is undetectable in metastatic breast cancer due to methylation of the *KLK6* gene promoter [70]. The gene CYP4F3 is part of a family of monooxygenases crucial for drug metabolism and lipid synthesis. Our results showed that calcitriol increased the expression of the *CYP4F3* gene both in the microarray and during validation by qPCR and western blot. Further studies are required to understand the significance of this result, as little is known about the role of the *CYP4F3* gene in cancer biology, except for a study suggesting that a potential single nucleotide polymorphism in this gene contributes to lung cancer progression, especially in smokers [71].

The anticipated interaction network derived from this study indicates the functional significance of the *CTNNB1* gene, which encodes catenin beta 1, along with the transcription factors S*OX2*, *NANOG*, and *ERG*. These proteins exhibit common traits including transcriptional regulation, participation in cellular proliferation and differentiation, and involvement in protein-protein regulatory networks [72–74]. Our analysis predicts that calcitriol inhibits these interaction networks in CaSki cells. While HPV is the primary etiological agent of cervical cancer, additional factors, including β-catenin dysregulation, significantly contribute to its progression [75]. β-Catenin, a pivotal component of the Wnt/β-catenin signaling pathway, plays a dual role in disrupting cell adhesion and signal transduction, thereby promoting carcinogenesis. In this study, predictive analysis using IPA software identified the CTNNB1 gene, as a key node within the interaction network of genes differentially regulated by calcitriol. However, microarray analysis in CaSki cells revealed no significant changes in CTNNB1 expression following calcitriol treatment. This observation suggests that calcitriol, via its receptor VDR, predominantly modulates the activity of the Wnt/β-catenin pathway rather than altering the expression of its components. In colorectal cancer, calcitriol suppresses cellular proliferation by inhibiting the Wnt/β-catenin pathway and enhancing E-cadherin expression, which reduces invasiveness. However, in this study, microarray analysis did not show calcitriol-induced changes in CDH1, the gene encoding E-cadherin. Notably, activated VDR interacts with β-catenin, preventing its association with TCF transcription factors [76]. This interaction inhibits β-catenin/TCF complex formation, reducing the expression of genes that promote cell proliferation and survival. This mechanism may explain the role of CTNNB1 as a central node in calcitriol-regulated networks. Interestingly, other key nodes like transcription factors SOX2, NANOG, and ERG also showed no differential expression. It is possible that calcitriol affects these factors through VDR-mediated modulation of their transcriptional activity [77]. Further research is needed to clarify the roles of these transcription factors in the antitumor effects of calcitriol in CaSki cells.

An interesting finding in our biological data analysis of CaSki cells is the observed negative regulation by calcitriol of the viral infection category, involving 125 implicated molecules. This finding corroborates prior evidence suggesting an association between vitamin D deficiency and its metabolites with the persistence of HPV DNA in early cervical cancer lesions [78]. Furthermore, a positive correlation has been identified between vitamin D levels and the persistence of 14 high-risk HPV subtypes [79]. Among the described antiviral mechanisms of calcitriol is the synthesis of antimicrobial peptides [4]. Interestingly, in this study, the *CAMP* gene, encoding the antimicrobial peptide cathelicidin, was significantly induced by calcitriol in CaSki cells, as previously described in other cell types [80,81]. Another relevant mechanism for the protective role of calcitriol against viral infections is the modulation of the inflammatory immune response [4]. As an example, in this study, the gene *S100A9* was induced by calcitriol in the microarray. S100A9 is a calcium-binding protein belonging to the S100 family, which plays a significant role in inflammation [82]. Furthermore, S100A9 is also an antimicrobial peptide with antiviral activity, which is exerted through different mechanisms, including interaction with the viral nucleocapsid and reverse transcription restriction, thus suppressing viral replication [83,84]. Even though S100A9 effects upon HPV infection in cervical cells remains to be elucidated, the heterocomplex S100A9/A8 induces apoptosis and inhibits metastasis of CaSki cells [85]. Together, these findings may explain the antiviral functions of calcitriol against high-risk HPV.

These results collectively suggest that modulation of gene expression by calcitriol extends beyond direct anticancer actions, influencing a wide array of biological and immunological processes. This comprehensive genomic response highlights the potential of calcitriol as a multifunctional agent in the therapeutic management of cervical cancer. Our findings also support the notion that an adequate nutritional status of vitamin D, whether through sunlight exposure, diet, or supplements, may help reduce the risk of cervical cancer and may have additional associated health benefits.

The relationship between calcitriol and HPV-related cancers, particularly cervical cancer, has received growing attention due to its promising therapeutic potential [4]. In cervical cancer, calcitriol exerts multiple anti-cancer effects, including the inhibition of cell proliferation through the downregulation of oncogenes such as HCCR-1 [46] and EAG1 [18], the latter being upregulated by high-risk HPV oncogenes [86]. Notably, the combination of calcitriol with cisplatin enhances its therapeutic efficacy, resulting in improved survival outcomes and suggesting a potential adjuvant role in the treatment of HPV-related malignancies [87]. Moreover, vitamin D metabolites have demonstrated the ability to inhibit cervical cancer progression and induce apoptosis through the activation of pro-apoptotic proteins and caspases [45]. Calcitriol also regulates the expression of specific microRNAs with anti-cancer properties. For instance, miR-22, which is downregulated by HPV16 E6 [88], can be restored by calcitriol [29], thereby inhibiting cancer cell proliferation and metastasis, although this effect was not observed in this study in CaSki cells. The effectiveness of calcitriol in treating HPV-positive cancers is influenced by several factors. For instance, a robust immune response against HPV enhances the efficacy of calcitriol [89,90]. While cervical cancer has been the primary focus of research, calcitriol also holds promise for other HPV-associated malignancies [89,90], including oropharyngeal, and vulvar cancers, indicating broader applications for calcitriol in the management of HPV-related cancers.

## Conclusions

Calcitriol significantly impacted the transcriptome of CaSki cells, an HPV16-positive cervical cancer model, regulating over 1000 protein-coding genes with a predominantly repressive effect. Validated findings included the downregulation of *EPHA2* and *RARA* genes and the induction of *KLK6* and *CYP4F3*. Calcitriol inhibited key tumor progression processes, such as cell proliferation and migration, and downregulated *MMP7* and *MMP13* mRNAs. Additionally, calcitriol modulated approximately 400 non-coding RNAs, including 111 microRNA precursors and 29 mature microRNAs, such as miR-6129, miR-382, miR-655, and miR-211, miR-590, miR-130a, miR-301a, and miR-1252, which are implicated in cervical cancer biology. These results indicate that calcitriol exerts significant antitumor effects by targeting critical cancer pathways, emphasizing the importance of maintaining adequate vitamin D levels.

## Supporting Information

S1 Table A complete list of differentially expressed genes by calcitriol was compared with vehicles in CaSki cervical cancer cells. The list includes protein coding sequences, long non-coding RNAs, and microRNA precursors.
(XLSX)

S2 Table Biological processes activated and suppressed, along with associated genes, induced by calcitriol treatment in CaSki cervical cancer cells. (XLS)

S1 Raw Images Uncropped original figures from the western blot experiments presented in this article. (PPTX)
