# Supplementary material for: Transcriptomic profile induced by calcitriol in CaSki human cervical cancer cell line
Source: PLoS One. 2025 Apr 1;20(4):e0319812. doi: 10.1371/journal.pone.0319812 (PMC11960991; doi:10.1371/journal.pone.0319812)
Supplement: S1 Raw Images — (PPTX) [file pone.0319812.s003.pptx]

## Slide 1
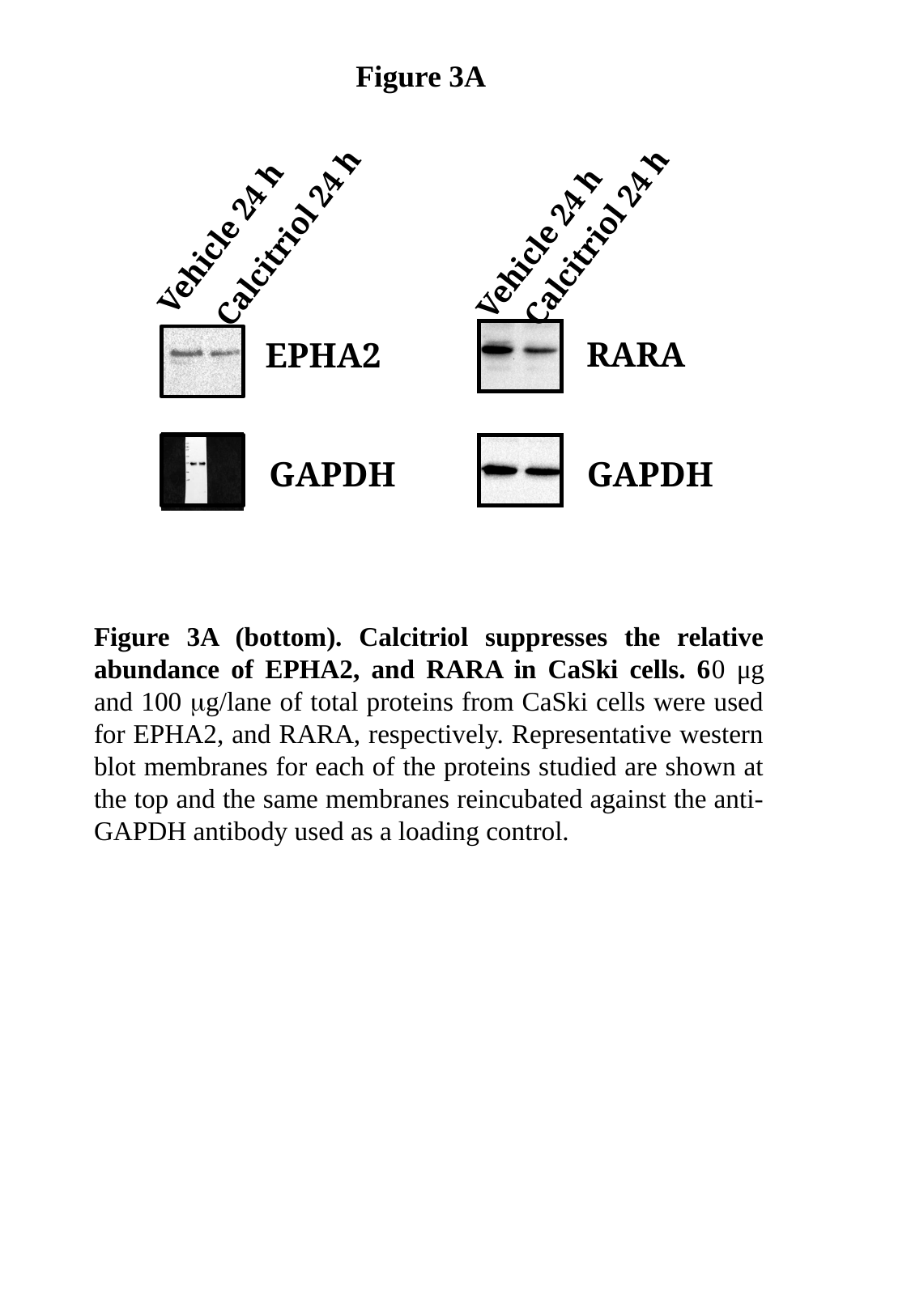

Figure 3A
Calcitriol 24 h
Calcitriol 24 h
Vehicle 24 h
Vehicle 24 h
RARA
EPHA2
GAPDH
GAPDH
Figure 3A (bottom). Calcitriol suppresses the relative abundance of EPHA2, and RARA in CaSki cells. 60 μg and 100 mg/lane of total proteins from CaSki cells were used for EPHA2, and RARA, respectively. Representative western blot membranes for each of the proteins studied are shown at the top and the same membranes reincubated against the anti-GAPDH antibody used as a loading control.

## Slide 2
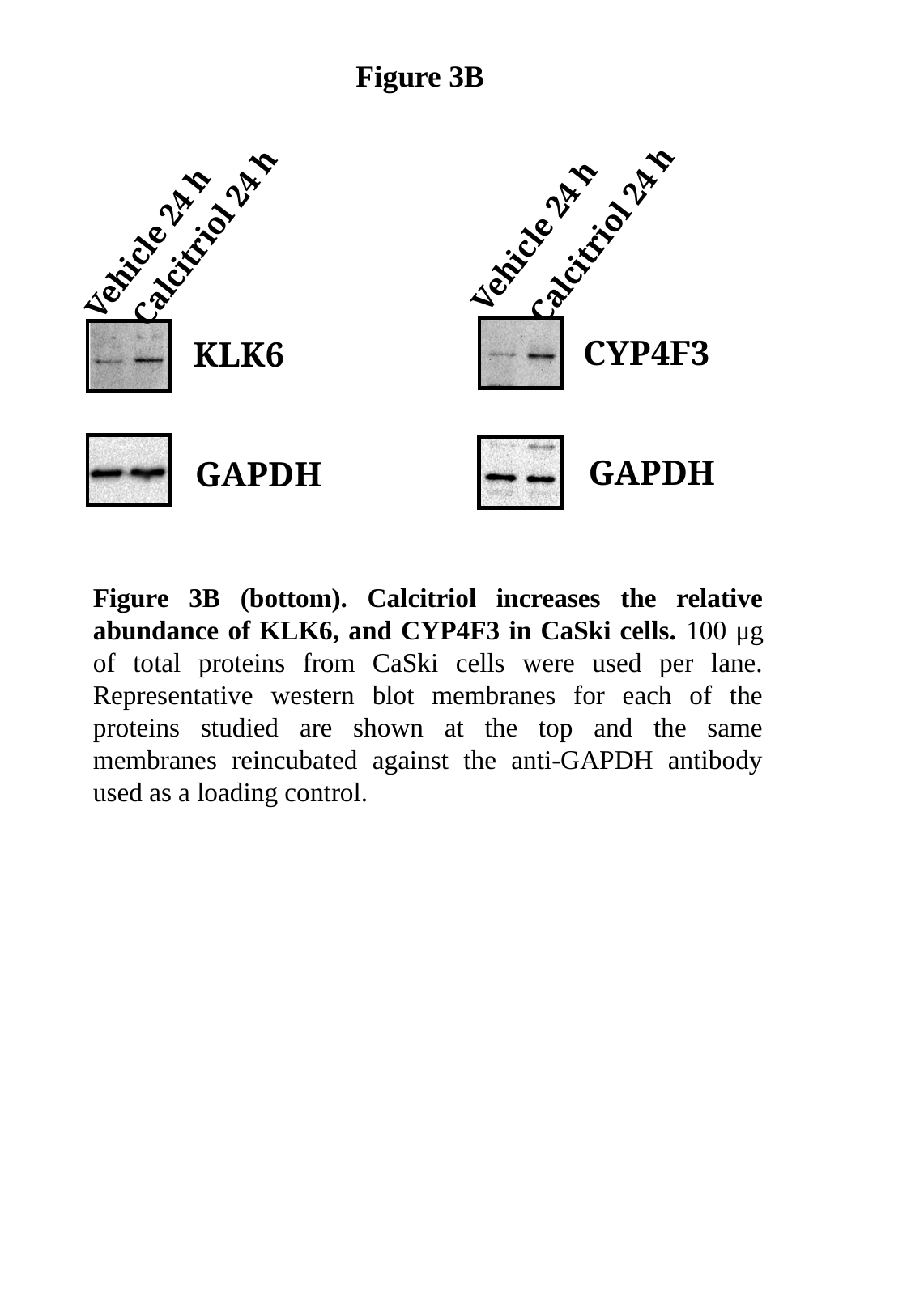

Figure 3B
Calcitriol 24 h
Vehicle 24 h
CYP4F3
GAPDH
Calcitriol 24 h
Vehicle 24 h
KLK6
GAPDH
Figure 3B (bottom). Calcitriol increases the relative abundance of KLK6, and CYP4F3 in CaSki cells. 100 μg of total proteins from CaSki cells were used per lane. Representative western blot membranes for each of the proteins studied are shown at the top and the same membranes reincubated against the anti-GAPDH antibody used as a loading control.

## Slide 3
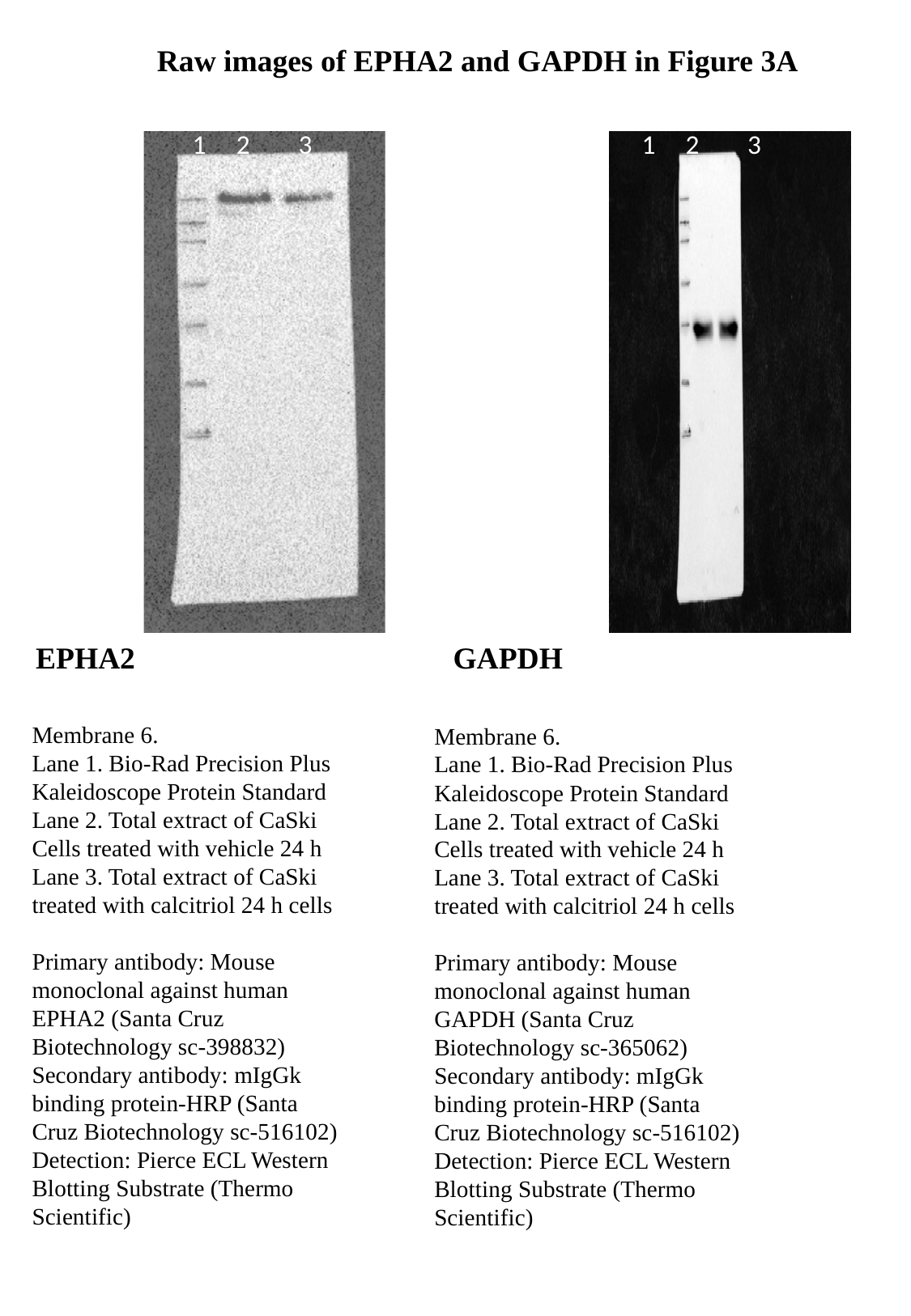

Raw images of EPHA2 and GAPDH in Figure 3A
1 2 3
1 2 3
EPHA2
GAPDH
Membrane 6.
Lane 1. Bio-Rad Precision Plus Kaleidoscope Protein Standard
Lane 2. Total extract of CaSki Cells treated with vehicle 24 h
Lane 3. Total extract of CaSki treated with calcitriol 24 h cells
Primary antibody: Mouse monoclonal against human EPHA2 (Santa Cruz Biotechnology sc-398832)
Secondary antibody: mIgGk binding protein-HRP (Santa Cruz Biotechnology sc-516102)
Detection: Pierce ECL Western Blotting Substrate (Thermo Scientific)
Membrane 6.
Lane 1. Bio-Rad Precision Plus Kaleidoscope Protein Standard
Lane 2. Total extract of CaSki Cells treated with vehicle 24 h
Lane 3. Total extract of CaSki treated with calcitriol 24 h cells
Primary antibody: Mouse monoclonal against human GAPDH (Santa Cruz Biotechnology sc-365062)
Secondary antibody: mIgGk binding protein-HRP (Santa Cruz Biotechnology sc-516102)
Detection: Pierce ECL Western Blotting Substrate (Thermo Scientific)

## Slide 4
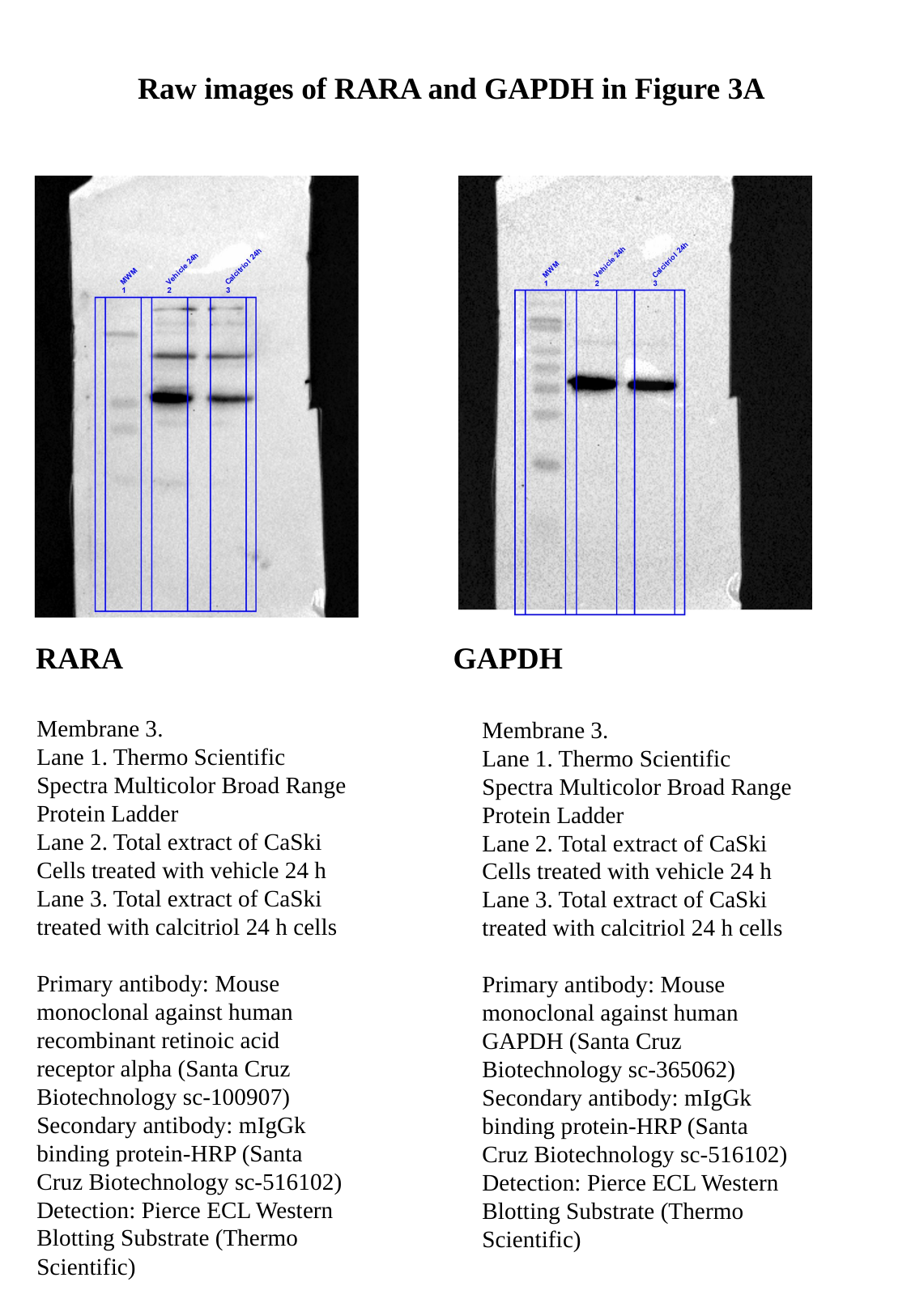

Raw images of RARA and GAPDH in Figure 3A
RARA
GAPDH
Membrane 3.
Lane 1. Thermo Scientific Spectra Multicolor Broad Range Protein Ladder
Lane 2. Total extract of CaSki Cells treated with vehicle 24 h
Lane 3. Total extract of CaSki treated with calcitriol 24 h cells
Primary antibody: Mouse monoclonal against human recombinant retinoic acid receptor alpha (Santa Cruz Biotechnology sc-100907)
Secondary antibody: mIgGk binding protein-HRP (Santa Cruz Biotechnology sc-516102)
Detection: Pierce ECL Western Blotting Substrate (Thermo Scientific)
Membrane 3.
Lane 1. Thermo Scientific Spectra Multicolor Broad Range Protein Ladder
Lane 2. Total extract of CaSki Cells treated with vehicle 24 h
Lane 3. Total extract of CaSki treated with calcitriol 24 h cells
Primary antibody: Mouse monoclonal against human GAPDH (Santa Cruz Biotechnology sc-365062)
Secondary antibody: mIgGk binding protein-HRP (Santa Cruz Biotechnology sc-516102)
Detection: Pierce ECL Western Blotting Substrate (Thermo Scientific)

## Slide 5
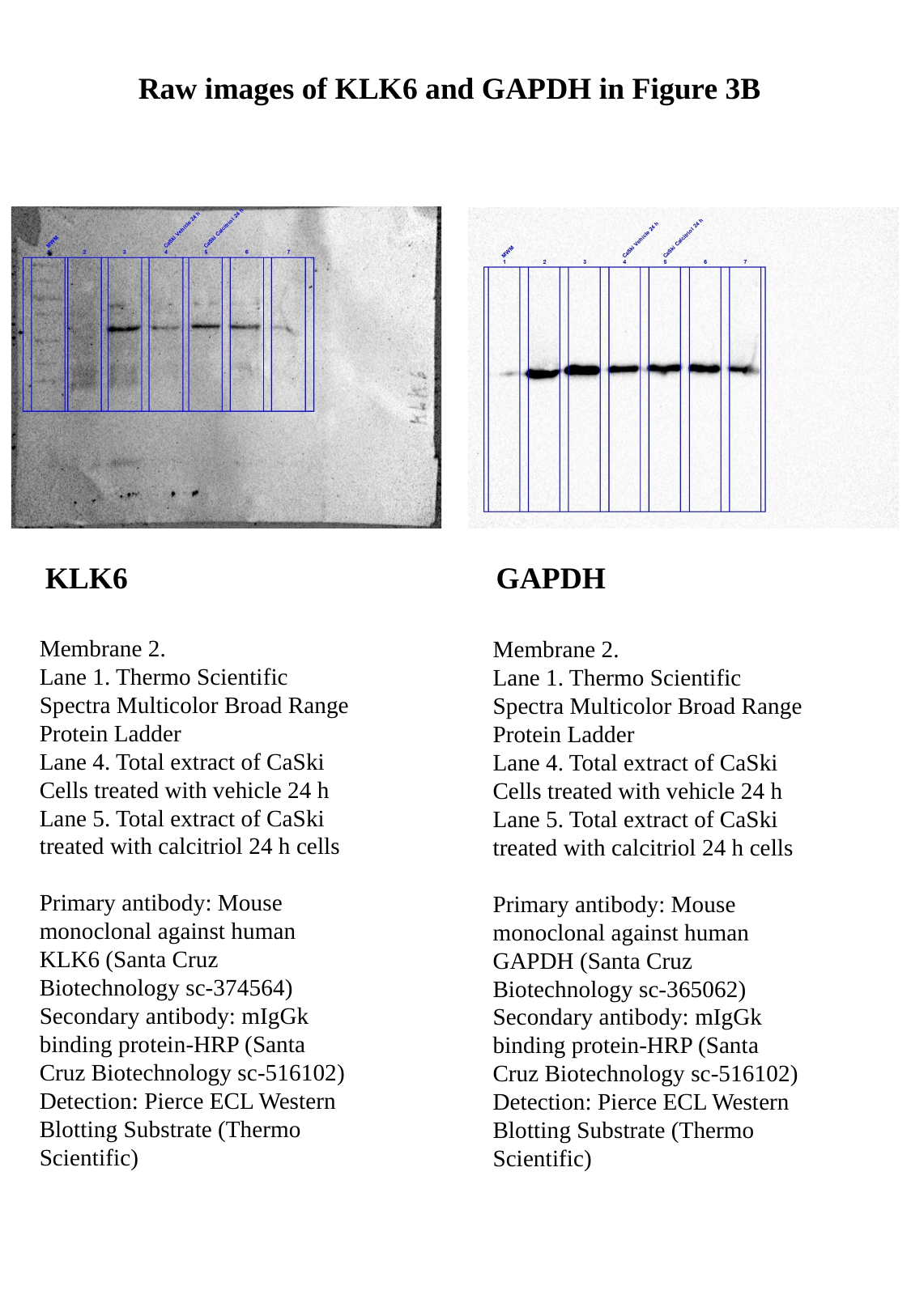

Raw images of KLK6 and GAPDH in Figure 3B
KLK6
GAPDH
Membrane 2.
Lane 1. Thermo Scientific Spectra Multicolor Broad Range Protein Ladder
Lane 4. Total extract of CaSki Cells treated with vehicle 24 h
Lane 5. Total extract of CaSki treated with calcitriol 24 h cells
Primary antibody: Mouse monoclonal against human KLK6 (Santa Cruz Biotechnology sc-374564)
Secondary antibody: mIgGk binding protein-HRP (Santa Cruz Biotechnology sc-516102)
Detection: Pierce ECL Western Blotting Substrate (Thermo Scientific)
Membrane 2.
Lane 1. Thermo Scientific Spectra Multicolor Broad Range Protein Ladder
Lane 4. Total extract of CaSki Cells treated with vehicle 24 h
Lane 5. Total extract of CaSki treated with calcitriol 24 h cells
Primary antibody: Mouse monoclonal against human GAPDH (Santa Cruz Biotechnology sc-365062)
Secondary antibody: mIgGk binding protein-HRP (Santa Cruz Biotechnology sc-516102)
Detection: Pierce ECL Western Blotting Substrate (Thermo Scientific)

## Slide 6
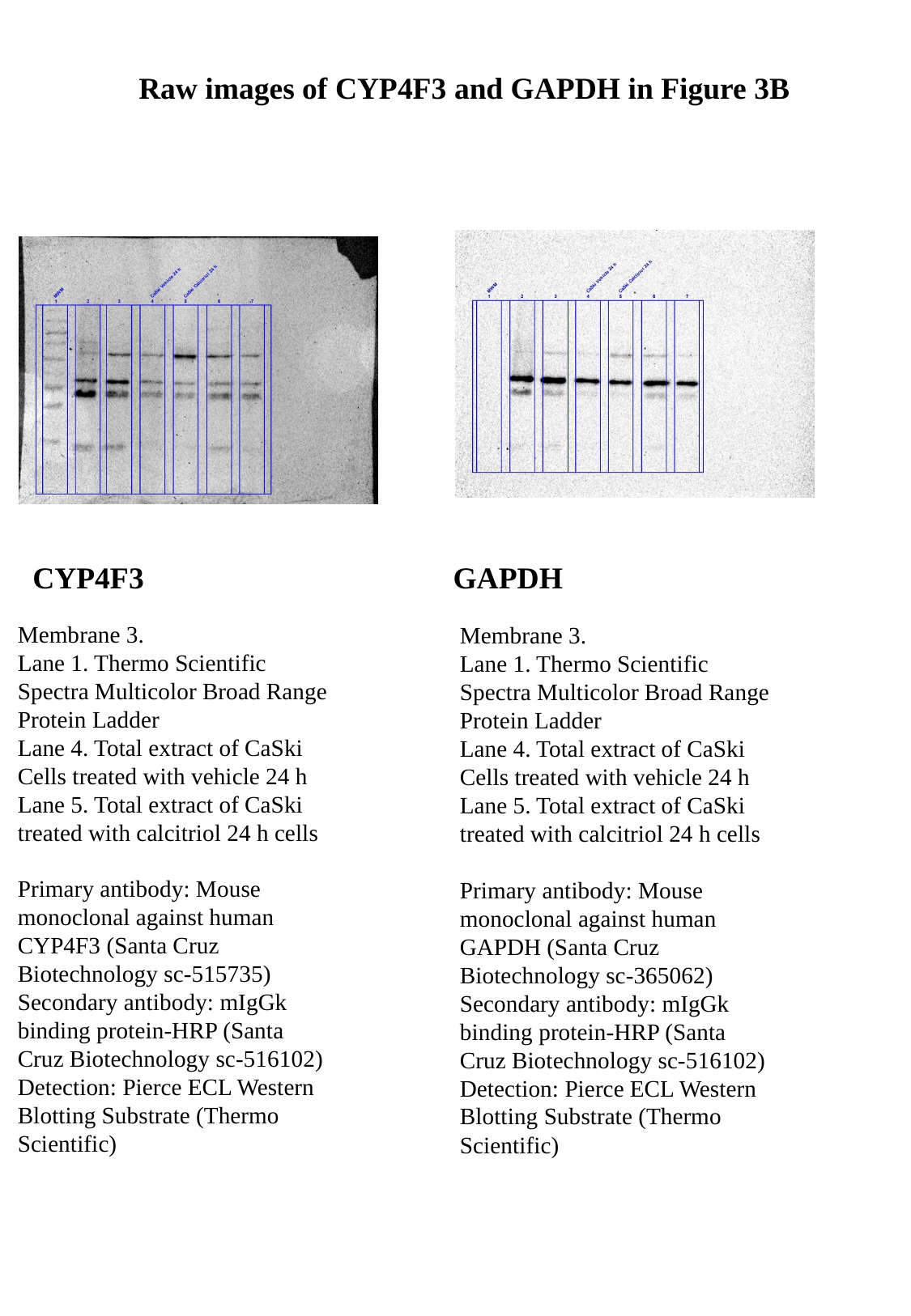

Raw images of CYP4F3 and GAPDH in Figure 3B
CYP4F3
GAPDH
Membrane 3.
Lane 1. Thermo Scientific Spectra Multicolor Broad Range Protein Ladder
Lane 4. Total extract of CaSki Cells treated with vehicle 24 h
Lane 5. Total extract of CaSki treated with calcitriol 24 h cells
Primary antibody: Mouse monoclonal against human CYP4F3 (Santa Cruz Biotechnology sc-515735)
Secondary antibody: mIgGk binding protein-HRP (Santa Cruz Biotechnology sc-516102)
Detection: Pierce ECL Western Blotting Substrate (Thermo Scientific)
Membrane 3.
Lane 1. Thermo Scientific Spectra Multicolor Broad Range Protein Ladder
Lane 4. Total extract of CaSki Cells treated with vehicle 24 h
Lane 5. Total extract of CaSki treated with calcitriol 24 h cells
Primary antibody: Mouse monoclonal against human GAPDH (Santa Cruz Biotechnology sc-365062)
Secondary antibody: mIgGk binding protein-HRP (Santa Cruz Biotechnology sc-516102)
Detection: Pierce ECL Western Blotting Substrate (Thermo Scientific)
